# Supplementary material for: Nature of the metal-insulator transition in few-unit-cell-thick LaNiO3 films
Source: Nat Commun. 2018 Jun 7;9:2206. doi: 10.1038/s41467-018-04546-5 (PMC5992201; doi:10.1038/s41467-018-04546-5)
Supplement: Supplementary file 1 — Supplementary Information [file 41467_2018_4546_MOESM1_ESM.pdf]

Supplementary Information

For

"Nature of the metal-insulator transition in few-unit-cell-thick $LaNiO_3$ films"

Golalikhani *et al*.

Supplementary Note 1: Oxide target preparation

To grow $LaNiO_3$ from separate oxide targets, we need La_2O_3 and NiO targets. NiO target was prepared by pressing and sintering high purity powder. For La_2O_3, the process is more complicated as La_2O_3 target is not stable in the ambient atmosphere due to quick hydration and carbonation of La_2O_3 upon exposure to air and moisture. To avoid this, the commercial La_2O_3 powder (purity 99.99%) was pre-sintered at 600°C for 24 hours as $La(OH)_3$ decomposes to La_2O_3 at this temperature. We then ground and pressed the powder into a dense pallet. The pallet was sintered at 1300°C for 24 hours in air and then cooled down to 300°C at 1°C/min cooling rate. After taking the target out of the furnace at about 200°C, it was transferred into vacuum chamber quickly and remained in vacuum.

Supplementary Note 2: $LaAlO_3$ buffer layer

Studies on the structure of 100 surfaces of $LaAlO_3$ single crystal shows that surface termination can be single LaO layer, single AlO_2 layer or the mixture of both depends on temperature and oxygen vacancies. Following the recipe by Ohnishi[1] AlO_2 terminated surface was achieved by HCl etching of single crystal substrate followed by annealing at 750°C in flowing oxygen. Supplementary Fig. 1a shows the Atomic Force Microscopy (AFM) image of the substrate after this treatment. The line profile of the scan shows flat traces with 1 u.c. high steps (Supplementary Fig. 1b). Homoepitaxial $LaAlO_3$ buffer layer (5-10 u.c.) was then deposited on the single terminated substrate in atomic layer by layer manner from La_2O_3 and Al_2O_3 targets. $LaAlO_3$ buffer layer were grown right before growth of $LaNiO_3$ film in the same growth chamber and identical growth parameters (oxygen pressure, substrate temperature, and laser spot size). Starting from the La_2O_3 target, a full atomic layer of LaO deposited followed by switching to Al_2O_3 target for the growth of a full AlO_2 atomic layer to complete 1 u.c. of $LaAlO_3$. We continue this until RHEED intensity oscillation shows the pattern characteristic of stoichiometry and full surface coverage. Supplementary Fig. 1c shows RHEED specular spot intensity oscillation during the growth of the last two $LaAlO_3$ layers followed by a layer of $LaNiO_3$. As it can be seen in the figure, an LaO layer brings RHEED intensity to a minimum while AlO_2 and

NiO_2 atomic layers bring RHEED intensity to maximize intensity. Sharp diffraction spots confirm the desired growth mode and surface quality (Supplementary Fig. 1d).

Supplementary Figure 1 Growth of homoepitaxial $LaAlO_3$ buffer layer. (a) An AFM scan of

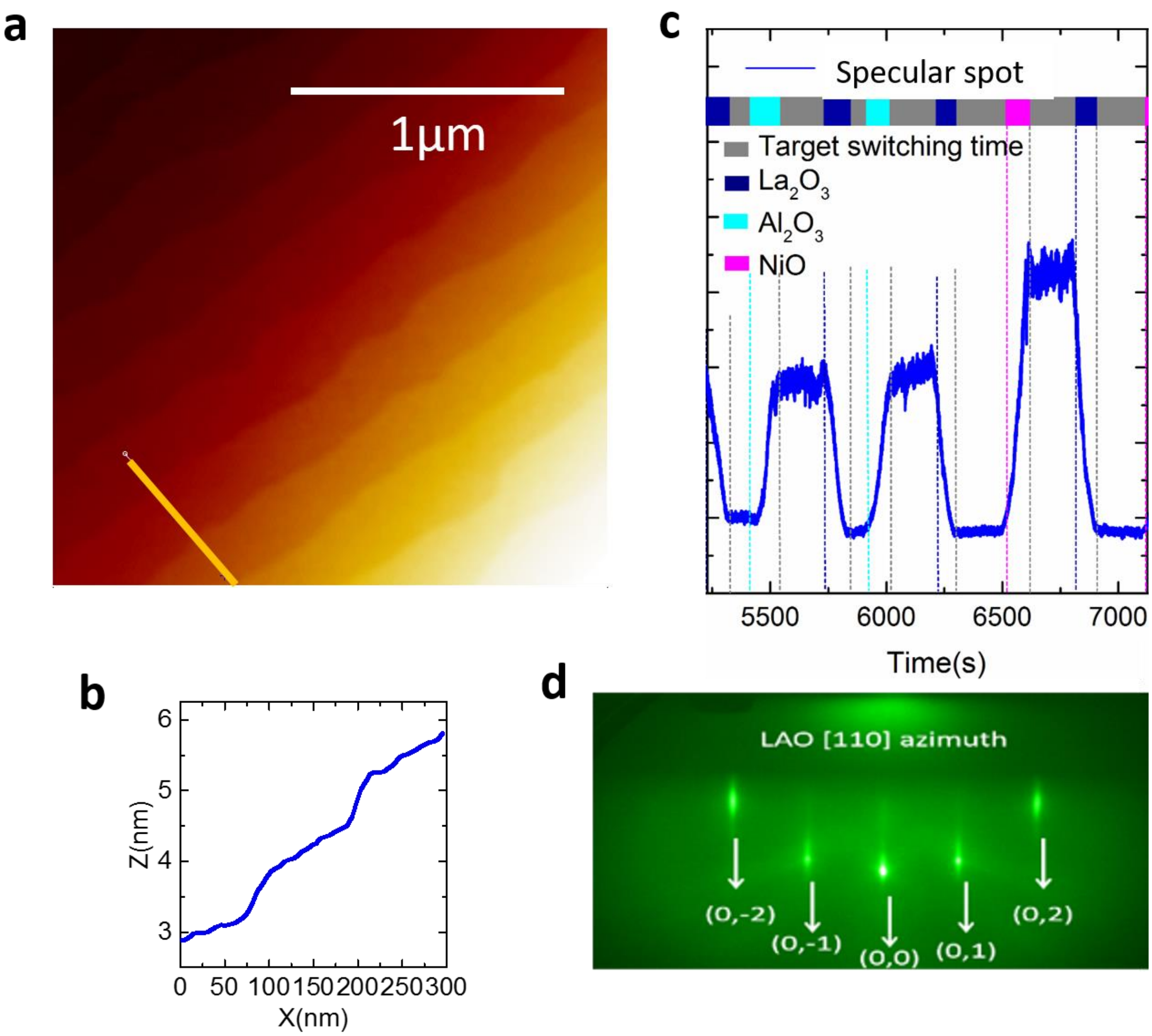

single terminated $LaAlO_3$ substrate. (b) Height profile of the orange line in (a) shows an atomically flat surface with 1 u.c. high steps indicative of single terminated substrate. (c) The RHEED specular spot intensity during the growth of 2 u.c. $LaAlO_3$ homoepitaxial layers followed by 1 u.c. of $LaNiO_3$ film. (d) The RHEED pattern after the growth of the homoepitaxial $LaAlO_3$ from Al_2O_3 and La_2O_3 targets. Strong and sharp spots are an indication of an atomically smooth surface

Supplementary Fig. 2 shows how the growth of $LaAlO_3$ buffer layer affects electrical properties of $LaNiO_3$ ultrathin films. Here we use LaO terminated samples with the thicknesses of 2 and 3 u.c. with and without $LaAlO_3$ buffer layer. For both thicknesses the buffer layer reduces the resistivity of the film, while for 2 u.c. sample this effect is more pronounced.

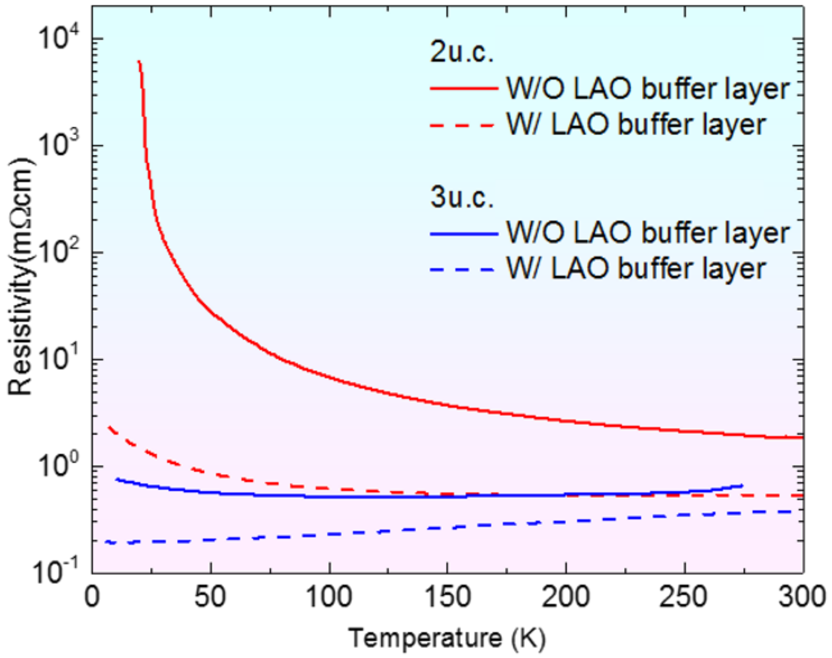

Supplementary Figure 2 Effect of $LaAlO_3$ buffer layer on electrical properties of the $LaNiO_3$ films. $LaAlO_3$ buffer layer reduces the resistivity of the 2 and 3 u.c. films compared to the ones grown under the same conditions but without the buffer layer. Influence of the buffer layer on electrical properties is larger for the thinner film.

Supplementary Note 3: $LaNiO_3$ growth and characterization

Epitaxial films of $LaNiO_3$ were grown on (001) pseudocubic $LaAlO_3$ substrates by Laser molecular beam epitaxy (MBE) equipped with reflection high-energy electron diffraction (RHEED). KrF excimer laser with 1-Hz pulse rate and 1-J/cm2 energy density was used to ablate oxide targets. Oxygen pressure of 7 Pa was used during the growth and the substrate was kept at a temperature of 650°C. After the growth, the substrate was maintained at the growth

temperature and additional oxygen was introduced into the chamber for post-annealing. This required increasing the heating power until the post annealing pressure of 8.5×10^4 Pa was reached. Films were post-annealed at this pressure for 30 min and then slowly cooled down to room temperature.

Following the growth of homoepitaxial $LaAlO_3$ buffer layer with an AlO_2 surface termination, a LaO layer will be first grown by ablating from a La_2O_3 target. RHEED intensity was monitored as the deposition took place so that the ablation was stopped when a full layer of LaO was deposited. The target will then be switched to NiO to grow the NiO_2 layer. Again, the RHEED intensity was monitored to ensure that one full layer of NiO_2 was deposited. The steps were repeated to grow a $LaNiO_3$ film of desired thickness and surface termination.

To calibrate the growth rate (number of pulses needed to complete one atomic layer), we first grow 40 u.c. $LaNiO_3$ film on $LaAlO_3$ substrate. The RHEED intensity oscillation was used as the primary tool to control the growth the mode. The as-grown 40 u.c. films were characterized by x-ray reflection for thickness measurement and by x-ray diffraction for the lattice constant measurement as well as for phase purity (Supplementary Fig. 3). Using the result of the $LaNiO_3$ film characterization, adjustments were made to the number of pulses if needed. As a more precise way to test the number of pulses required for La-Ni stoichiometry, we used the number of pulses necessary for each atomic layer of LaO and NiO_2 from the $LaNiO_3$ calibration to grow La_2NiO_4, the Ruddlesden-Popper phase with $n = 1$. It has been shown that the growth of the Ruddlesden-Popper phase is more sensitive to stoichiometry and full layer coverage. We use the ablation sequence of La_2O_3- La_2O_3-NiO for each u.c.

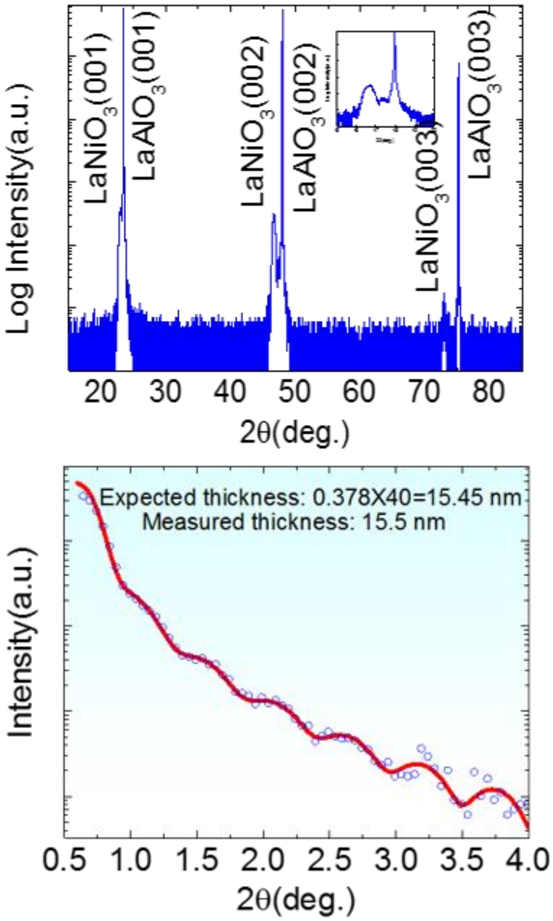

Supplementary Figure 3 Characterization of a 40 u.c. $LaNiO_3$ film. (a) X-ray diffraction (XRD) for a 40u.c. $LaNiO_3$ grown on $LaAlO_3$ substrate showing single phase epitaxial growth. (b) X-ray reflection (XRR) for a 40u.c. $LaNiO_3$ grown on $LaAlO_3$ substrate. The measured thickness (15.45 nm) is in close agreement with the expected thickness (15.5 nm).

Supplementary Fig. 4 shows RHEED intensity oscillation and XRD θ-2θ scan for the growth of La_2NiO_4 film. The persistent RHEED oscillation confirms the atomic layer-by-layer growth mode and the sharp peaks in XRD scan shows the phase purity of our film. We found out that even with the most precise calibration, *in-situ* monitoring and control were needed for each film.

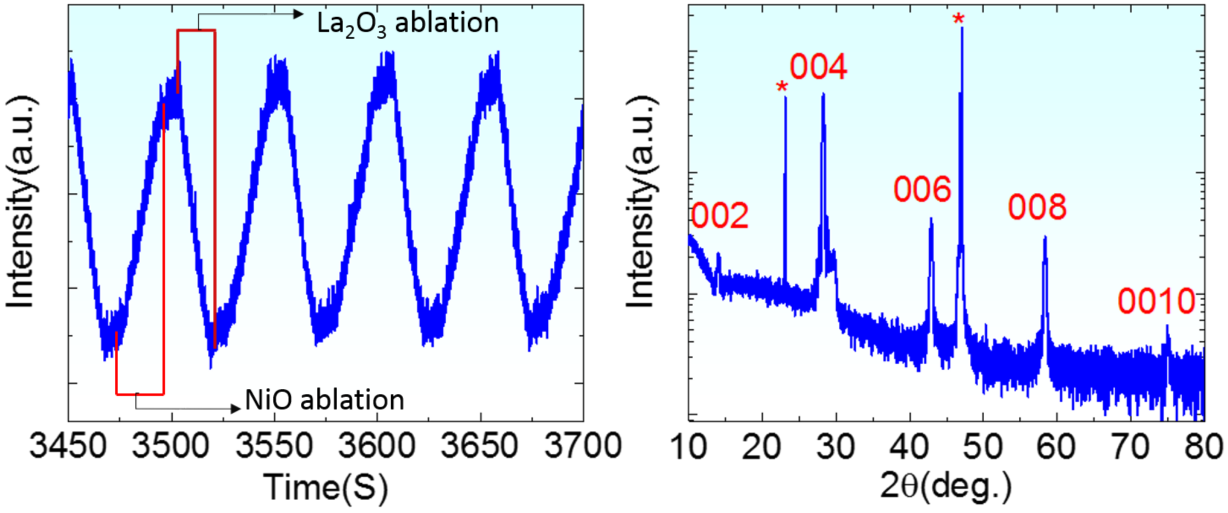

Supplementary Figure 4 Growth and characterization of La_2NiO_4. (a) RHEED intensity oscillation during the growth and (b) X-ray diffraction (XRD) for Ruddlesden-Popper $La_{n+1}Ni_nO_{3n+1}$ with n = 1 on LSAT substrate.

We found that the transport properties of $LaNiO_3$ films were strongly dependent on the concentration of oxygen vacancies in the films. To reduce the oxygen vacancies, oxygen pressure was set to the maximum achievable in our chamber without affecting the RHEED spot quality. We have also investigated the effect of post annealing oxygen pressure on the transport properties of the films. Supplementary Fig. 5 shows the temperature dependent resistivity for 1.5 u.c. films grown at the same condition but post-annealed at 2 different oxygen pressures. We can see a slight improvement in transport properties of the film post-annealed at a higher oxygen pressure.

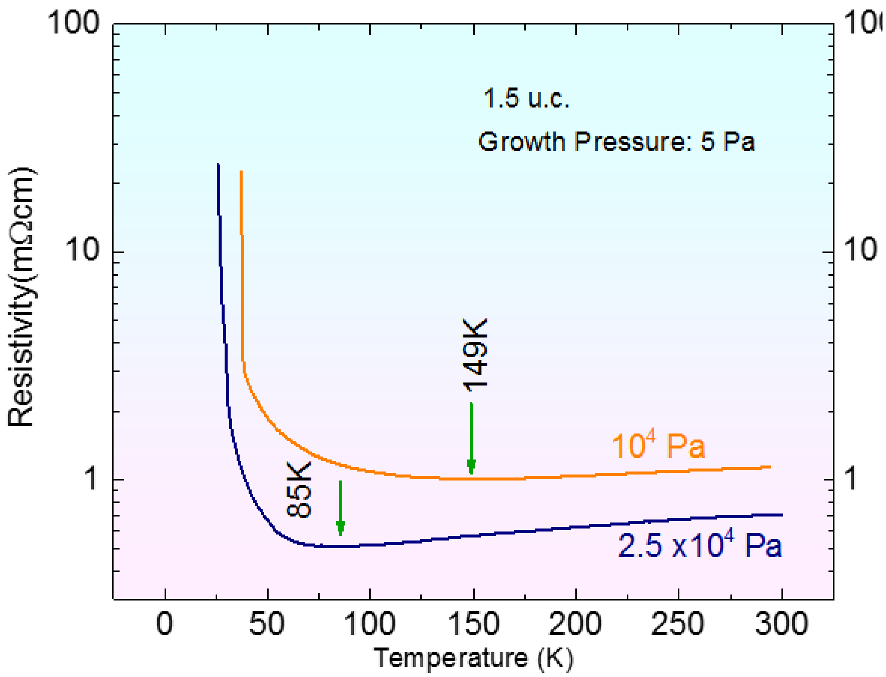

Supplementary Figure 5 effect of post-annealing pressure on film's transport properties. Resistivity vs. temperature for 2 u.c. NiO_2 terminated $LaNiO_3$ films grown under the same condition but post-annealed at two different pressures. Both room temperature resistivity and the MIT temperature decreased as the post-annealing pressure increased.

Supplementary Fig. 6 shows orbital polarization in $LaNiO_3$ films. XAS spectra at Ni L_2-edge for $\mathbf{E}\perp c$ (red) and $\mathbf{E}\| c$ (black) polarizations are shown for $LaNiO_3$ films with seven different thicknesses..

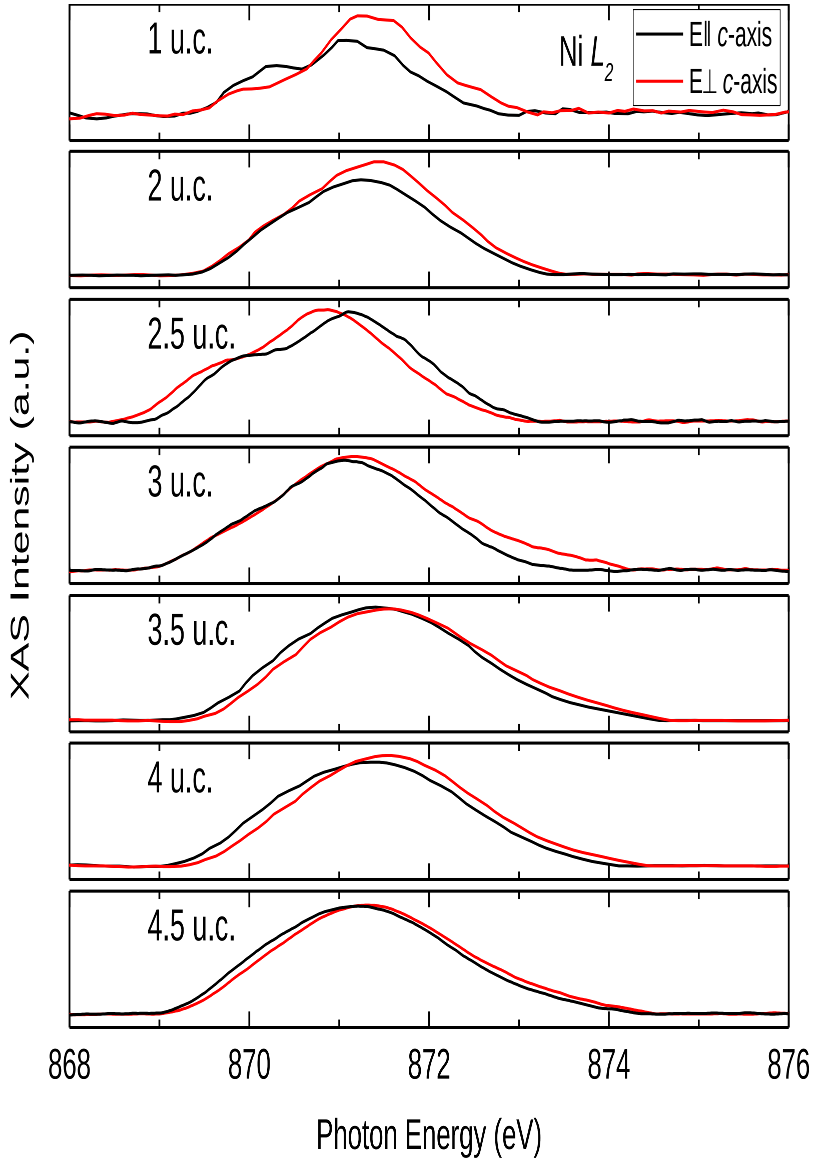

Supplementary Figure 6, Orbital polarization in the $LaNiO_3$ films. XAS spectra at Ni L_2-edge for **E**⊥*c* (red) and **E**∥*c* (black) polarizations $LaNiO_3$ films with different thicknesses.

Supplementary References

1. Ohnishi, T. *et al.* A-site layer terminated perovskite substrate: $NdGaO_3$. *Appl. Phys. Lett.* **74,** 2531 (1999).
